# Forensic tool for sex prediction- hand dimensions

**DOI:** 10.4314/ahs.v22i4.46

**Published:** 2022-12

**Authors:** Renu Gupta, Ashish Kumar Nayyar, Manoj Kumar Gupta, Om Lata Bhagat

**Affiliations:** 1 All India Institute of Medical Sciences Jodphur, Department of Anatomy; 2 All India Institute of Medical Sciences Jodphur, Department of Community Medicine and Family Medicine; 3 All India Institute of Medical Sciences Jodphur, Department of Physiology

**Keywords:** hand measurements, sexual dimorphism, demarking point, discriminant analysis

## Abstract

**Background:**

Determination of sex from mutilated body fragments can perform vital role for identification of departed soul. Forensic authority pacts with human identification from the hand measurements which is of prodigious assessment during tragedies, terror attacks and in criminality.

**Objective:**

Present study explored the analytical role of the anthropometric measurements of hand dimensions, find demarking points for male and female, check percentage exactitude of sex determination in Western Indian population.

**Methods:**

The study was piloted on a sample of 504 individuals. All the measurements were taken by standard procedure. Discriminant analysis and demarking points were created for all hand measurements.

**Results:**

Males have a significantly higher values of all measurements than females. The left-hand length measurement unveiled a noteworthy sexual dimorphism index (110.80). The higher value of the demarking point labelled as males. The best sexually dimorphic hand dimensions showed the utmost precision left hand length (95% in the male), followed by right hand length (76.1%).

**Conclusion:**

All hand measurements like length, breadth and index are displaying sexual dimorphism, hence they can be used for determination of sex when isolated hand is found.

## Introduction

The most vital stage in medico legal practices is accurate identification of individual. The establishment of the individuality of the departed soul in cases of catastrophe is challenge to the forensic experts 1. In contemporary period, disjointed body fragments are repeatedly found as a result of increased procedures of natural and man-made tragedies and also owing to augmented trials of the assassinations where the disfigurement of deceased is done by a slayer to rescind all traces of individuality in addition to ease of the disposal of the dead. Forensic anthropologist can deliver a timid credentials of mysterious residues by framing a ‘biological profile’, which comprises the determination of stature, sex, age and ethnicity in such cases 2. Sex determination is frequently a modest chore in forensic inquiry when entire body is existing by external or internal genitalia; but become problematic while disarticulated body shares are found. Former anthropologists had to be reliant solely upon pelvic and skull bones to determine the sex but now there is inclination towards long bone as well as body parts like hand 3,4,5. With the innovation of recent expertise, DNA analysis for determination of sex is much modest and easier way. But several times it cannot achieves the anticipations to identify mutilated or scrappy residues, apart from that it cannot be working in all the cases due to lack of trained human resources, time consumption and restricted spending 6. Therefore, anthropometry is still frequent choice for identification of individuals 7.

Anthropometric measurements of different populations are different due to inherited and ecological factors; hence population specific demarking point (cut off point) should be created 8. For that reason, in present study demarking points for Hand dimensions was derived in Western Indian population.

## Material and methods

A cross sectional descriptive study was done on 504 healthy individuals (male 308 and female 196) of age-group 22- 40 years from western India. The people having any history of injuries and deformity in hand were omitted from the study. Harpenden anthropometry set was used for all the measurements, expressed in centimetre and taken by the same individual to minimize inter-observer error.

### Measurements

**Figure 1:**
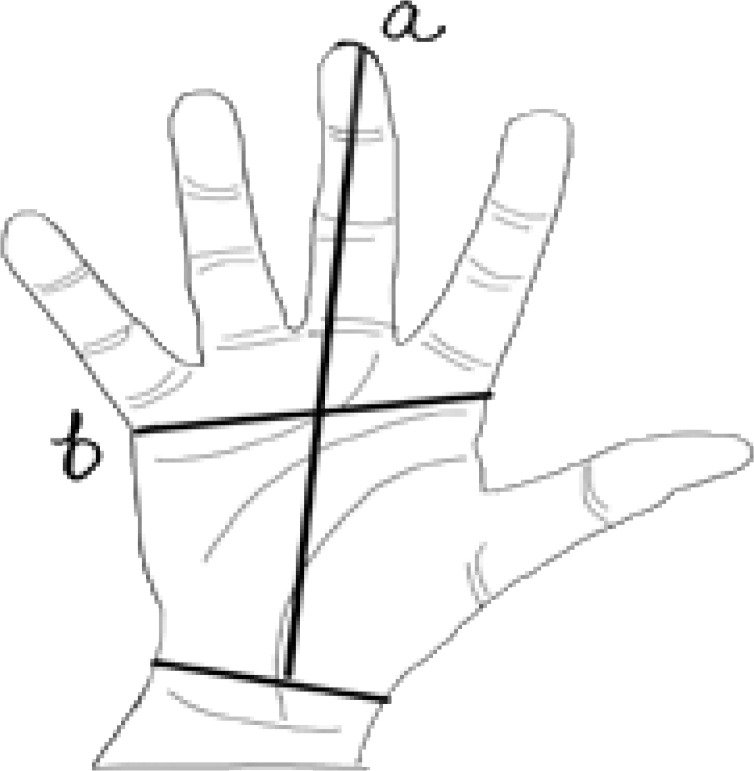
Diagrammatic representation of (a) Distance from the mid-point of the distal crease of the wrist joint to the most anterior projecting point on the tip of the middle finger as hand length. (b) Distance from the most laterally placed point on the head of the 2nd metacarpal to the most medially placed point located on the head of the 5th metacarpal as hand breadth. Adapted from: Gupta et. al. Mymensingh Med J 2020; 29 (3): 709–719.

Hand length: The distance from the mid-point of the distal crease of the wrist joint to the most anterior projecting point on the tip of the middle finger were recorded as hand length (figure 1).

Hand breadth – For hand breadth, distance from the most laterally placed point on the head of the 2nd metacarpal to the most medially placed point located on the head of the 5th metacarpal was taken. (Figure 1).

Hand index was calculated by dividing hand breadth with the hand length and multiplied by 100.

### Statistical analysis

The obtained data were calculated and evaluated by SPSS 21. Descriptive statistics like mean, SD were expressed for all hand dimension. The hand dimension between sexes were compared by independent sample t-test, and the level of statistical significance was set at P < 0.05. For accuracy of sex determination, the discriminant analysis was used and a demarking point for each variable was calculated. The demarking point, was calculated by taking average of the mean value of each sex and accordingly males were categorized with greater values and females with lesser values of demarking point.

A cross-validation method “leave one out classification” technique was applied to the model to quantify the precision of the cataloguing of the original and the one generated by cross validation. More precise outcomes attained in the cross-validation point out greater reliability of the discriminant function.

## Results

Descriptive statistics for hand measurements of both sexes are presented in Table 1. It was observed that males have a significantly higher value than females. These findings in the direction of the presence of sexual dimorphism in the study model. The sexual dimorphism index was calculated by dividing the male mean by the female mean and multiplied by 100. The value of sexual dimorphism index more than 100 better for sex estimation. The left-hand length measurement exhibited a significant degree of sexual dimorphism (110.80) (Table no 1).

**Table 1:** Descriptive statistics of hand measurements

Parameters	Male (Mean± SD)	Female (Mean±SD)	f value	Significance (<0.001)
RHL	16.89 ± 1.02	15.83 ± 0.74	159.170	.000
RHB	8.33 ± 0.71	7.97 ± 0.42	41.465	.000
LHL	18.05 ± 2.52	16.29 ±1.72	73.025	.000
LHB	8.35 ± 0.69	7.95 ± 0.45	50.165	.000
RHI	49.34 ± 0.11	50.31 ± 0.51	89.31	.000
LHI	46.86 ± 0.45	49.10 ± 0.68	45.68	.000

* Canonical correlation = 0.851, Wilk lambda = 0.276 and Box's M (p = < 0.001) RHL= Right hand length, RHB= Right hand breadth, LHL= Left hand length, LHB = Left hand breadth, RHI = Right hand Index, LHI = Left hand Index

Discriminant analysis and demarking points were generated for the hand measurements (Table 2). The higher value of the demarking point labelled as males.

**Table 2:** Stepwise discriminant function analysis for sex determination by hand measurements

Parameters	Wilk's lambda	Canonical correlation	Structure matrix	Demarking point
RHL	.760	-.255	.347	F < 16.36 < M
RHB	.924	-.165	.177	F < 08.15 < M
LHL	.873	-.041	.235	F < 17.17 < M
LHB	.909	.056	.195	F < 08.15 < M
RHI	.836	-.154	.298	F < 49.85 < M
LHI	.912	.096	.185	F < 47.98 < M

RHL= Right hand length, RHB= Right hand breadth, LHL= Left hand length, LHB = Left hand breadth, RHI = Right hand Index, LHI = Left hand Index, M= male, F= female.

The range of classification accuracy was between 30 – 95% perceived (table no 3). The most sexually dimorphic hand measurements showed the highest accuracy left hand length (95% in the male), followed by right hand length (76.1%). Multivariate and cross-validation classification using “leave-one-out” classification method was used for all the calculations. It was also observed by using stepwise analysis that the classification accuracy was increased when all measurements were taken together (the original value 95.2% and cross-validation value was 94.7%) (Table no 3).

**Table 3:** Classification accuracy expressed as percentage in hand measurements

Parameters	Expected accuracy (Male)		Expected accuracy (Female)		Expected accuracy (total)	
	Original	Crossvalidated	Original	Crossvalidated	Original	Crossvalidated
RHL	76.1	75.7	75.5	75.5	75.8	75.6
RHB	74.8	74.8	31.6	30.6	58.0	57.6
LHL	95.1	95.1	65.8	65.8	83.8	83.8
LHB	77.3	77.3	45.4	45.4	65.0	65.0
All measurements	95.1	94.5	95.4	94.9	95.2	94.7

RHL= Right hand length, RHB= Right hand breadth, LHL= Left hand length, LHB = Left hand breadth

Receiver operating characteristic (ROC) curve for hand dimensions in both the genders to represent the discriminative power of the ratios by the area under the curve (AUC). It was observed areas under curve in right arm breadth 0.648, left palm length 0.787 and left palm breadth 0.674 in males whereas in female AUC in right palm breadth 0.811, left palm length 0.787 and left palm breadth 0.674 (Figure no 2). It is evident from the ROC testing used on the exactitude possibility that both right and left hands dimensions have sex discrimination capabilities.

**Figure 2 (a):**
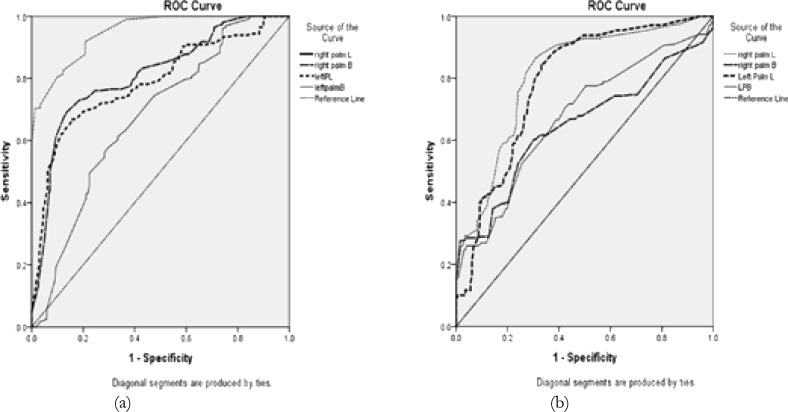
Receiver Operating characteristic (ROC) Curve representing sensitivities, specificities of the all hand dimensions in females. (Value of area under the curve (AUC) for right palm length: 0.948) (b) Receiver Operating characteristic (ROC) Curve representing sensitivities, specificities of the all hand dimensions in males. (Value of area under the curve (AUC) for right palm length: 0.811)

## Discussion

During any mishaps, bomb explosions, natural disasters, crime investigations, and ethnic studies, Sex determination turn out to be the earliest precedence in the process of identification of a person 9. The human hand is the most used portion of the physique which can used to determine stature 10. The stature and sex may help in identification; therefore, this study was conducted to find out if hand dimensions and their indices can be used to determine sexual dimorphism 8.

In present study hand dimensions of male (hand length and breadth) were found to be higher as compared to the female hand dimensions. No arithmetical noteworthy differences were observed in the length and breadth of the right and left hands. This end result is in harmony with study of Ibrahim et al. 11 Dey et al. 12 Pandeya et al. 13 and many more previous studies 14, 15. Earlier union of bones is probable ground of lesser dimensions in females.

Hand Length & Breadth are reliant on body dimension of the individual hence hand index was calculated in the current study to combat as it is self-regulating and not associated to stature as well as age and more consistent to determine the sex. The average hand index of male was 49.34 in right and 46.86 in left hand while in females, the average hand index was 50.31 in right and 49.10 in left hand. Male-female variances were found statistically significant at p<0.001 for both right and left hand however non-significant for dissimilarity between right- and left-hand index in both sexes. This is in agreement with the conclusions perceived by different studies. 16,17

Value of demarking point acquired by this study is greater than that found in north and south Indian population and in Egyptian population 17,18,19,20, whereas lesser than that obtained by Varu PR et al. 8. Impact of race and ethnicity effects in divergence of hand dimensions that might have directed to diverse values of hand index for male and female. A fundamental opinion is that these dimensions should be done independently in each population, since the ethnic dissimilarities are real on these measures and condense the leeway of generalizing.

Sex determination may be done by a morphological approach of hand measurements.
